# Real-Time Dry Matter Prediction in Whole-Plant Corn Forage and Silage Using Portable Near-Infrared Spectroscopy

**DOI:** 10.3390/ani15162349

**Published:** 2025-08-11

**Authors:** Matheus Rebouças Pupo, Evan Cole Diepersloot, Eduardo Marostegan de Paula, João Ricardo Rebouças Dórea, Lucas Ghedin Ghizzi, Luiz Felipe Ferraretto

**Affiliations:** 1 Department of Animal and Dairy Sciences, University of Wisconsin—Madison, 1675 Observatory Dr., 934F Animal Sciences Building, Madison, WI 53706, USA; 2 Department of Animal Sciences, University of Florida, Gainesville, FL 32608, USA

**Keywords:** Koster, moisture, oven drying, scanning methods

## Abstract

Portable near-infrared reflectance spectroscopy (NIRS) offers the opportunity of a rapid measurement of forage dry matter concentration, allowing producers to make faster adjustments in real time. This study compared dry matter (DM) concentration predictions of three units of a portable near-infrared reflectance spectrometer (pNIRS) with conventional forced-air oven drying (48 h at 60 °C) using corn forage and silage samples. Moreover, a common on-farm method (Koster tester) was also compared. The reflectance curve used by pNIRS to predict DM was obtained by scanning WPCS samples and developed by SciO. A total of 113 whole-plant corn forage (WPCF) and 27 whole-plant corn silage (WPCS) samples from 66 corn hybrids were obtained from three separate experiments conducted between 2018 and 2019. These three experiments were completely independent of each other, with sample collections over different periods. In Experiment 1, all treatments were tested in WPCF, and the DM concentration of the forced-air oven differed from Koster testers (35.4 vs. 34.3% DM, on average, respectively) and all three pNIRS units (35.4 vs. 30.7% DM, on average, respectively), with no differences among pNIRS. All treatments were positively correlated with the forced-air oven treatment DM values. Experiment 2 evaluated the Koster tester and pNIRS in WPCF on farms, and the Koster tester differed from pNIRS (37.2 vs. 33.3% DM, on average, respectively) treatments. All pNIRS were positively correlated with Koster tester treatment. In Experiment 3, all treatments were tested in WPCS, and the DM concentration of the forced-air oven was greater than other treatments (35.3 vs. 32.8% DM, on average, respectively). Overall, Koster tester predictions for both Experiments 1 and 3 were better correlated with the forced-air oven than pNIRS. Additionally, pNIRS showed a lower mean bias, although low coefficients of determination and concordance correlation were observed in Experiment 3 compared to Experiments 1 and 2, which might be related to the prediction curve. Further calibrations of the predictive curve with forage samples would be needed to reasonably estimate the DM concentration of WPCF, whereas a greater number of samples could account for the variations in WPCS due to large heterogeneity in particle composition (e.g., leaves, stem, and kernel), size, and distribution.

## Simple Summary

Portable near-infrared spectroscopy (pNIRS) devices continue to gain popularity among dairy producers and consultants, enabling rapid determination of on-farm feed dry matter concentrations. However, further evaluation of the variation within handheld pNIRS units and their comparison with forced-air oven and Koster tester DM measurements are needed. The objective of this study was to (1) evaluate the variation within handheld pNIRS units and (2) compare pNIRS with forced-air oven and Koster tester DM measurements. A total of 113 whole-plant corn forage (WPCF) and 27 whole-plant corn silage (WPCS) samples from 66 corn hybrids were obtained from three separate experiments conducted between 2018 and 2019. Across the three experiments, forced-air oven and Koster tester treatments provided consistent DM values, while pNIRS units tended to predict lower DM values (~4 percentage units). In this current study, differences between pNIRS and other traditional methods may be related to the calibration model being created solely with ensiled samples, whereas the experiment used unfermented samples. Portable NIRS might be a viable option on farms for time-sensitive measurements with an appropriate prediction curve. By enabling rapid and reliable DM assessments, pNIRS units could help farmers optimize feed quality, improve livestock productivity, and make more informed decisions regarding forage management. Further research to evaluate the use of pNIRS for determining DM concentration in other forages and feeds is warranted.

## 1. Introduction

Silages often comprise over half of the dry matter (DM) portion of lactating dairy cow diets [1], and variation in silage DM concentration or nutritive value impacts nutrient composition of the total mixed ration, impairing animal performance. Dry matter concentration of corn silage may vary substantially over short periods of time because of growing conditions, maturity at harvest, storage method, silo management, and environmental conditions affecting silo surface exposed to air [2,3]. For example, the average range in DM concentration of monthly corn silage samples within a farm (over a 12-month period) varied by up to 9 percentage units, whereas daily samples over a 14-day period varied by 7 percentage units in a study by [4]. Additionally, changes in silage DM concentration ranging from 5 to 10 percentage units have been reported during the summer season depending on the type of storage structure, length of surface exposed to the air, and type of silo [2]. Thus, measuring the extent of DM variation within a farm could be used to determine (1) adjustments to delivered dairy diets on a farm, (2) whether a forage source must be re-tested for nutrient analysis, and (3) whether a forage source should be tested several times. Inconsistency in nutrient composition of delivered diets can affect the performance of lactating cows [5]. Monitoring DM concentration prior to and during harvest is also vital because it provides real-time information for farmers or custom harvesters about plant maturity, allowing for real-time adjustments in harvester settings. Additionally, DM concentration at harvest affects fermentation patterns, effluent production, packing density, and aerobic deterioration during ensiling [6]. Thus, it is imperative to evaluate the DM of whole-plant corn forage (WPCF) quickly and accurately on farms to achieve targeted silage DM and manage harvest constraints related to time, cost (i.e., collection of representative samples), and inclement weather [7,8].

However, on-farm DM measurement may be time-consuming or inaccurate [9]. One of the most accurate and widely accepted methods to determine DM concentration is drying samples in a forced-air oven [10] under laboratory conditions. Nevertheless, this approach is not widely adopted on farms because of the costs and time required for DM determination (i.e., 48 h at 60 °C). Instead, other air-drying systems, such as Koster testers, are often used. However, previous studies reported high variability and low accuracy of Koster tester systems depending on the sample type [9]. The total error of Koster testers compared to the forced-air oven ranges from 6.8 to 9.4 percentage units, on average [9,11]. Using portable near-infrared spectrometers (pNIRS), another common DM measurement method used on farms recently, has been reported to be more rapid, effective, and reliable for forage DM determination than alternative techniques (i.e., electronic nose, X-ray fluorescence) [12,13]. Recent developments in smaller and more portable versions of this technology enabled rapid determination of on-farm DM concentration to evaluate both forages and silages [14,15,16]. The performance of handheld NIR instruments can be comparable to that of laboratory NIR instruments for dried-ground samples [17,18,19,20]. Likewise, ref. [21] indicated that handheld NIR could accurately predict DM concentration of haylages using the mixed silage calibration. Although handheld NIR instruments have shown the ability to predict DM concentration as well as laboratory-based NIR instruments in a wide range across the NIR spectrum [22], little is known about their accuracy with a narrow NIR scan. Therefore, the primary objective of this study was to evaluate the variation within handheld pNIRS units and compare pNIRS with forced-air oven and Koster tester DM measurements. A secondary objective was to evaluate whether a calibration model developed using whole-plant corn silage (WPCS), varying in moisture content and particle size, could effectively predict the DM concentration of WPCF. Our hypothesis was that a pNIRS model calibrated using WPCS would estimate DM concentration similarly to the forced-air oven method. Additionally, our hypothesis was that this model would also accurately predict the DM concentration of WPCF similarly to the forced-air oven method.

## 2. Materials and Methods

### 2.1. Handheld Units and Scanning Procedures

The handheld pNIRS units (wavelength: 740–1070 nm; weight: 35 g; size: 3.15 × 9.5 × 27.5 mm) manufactured by SciO (Consumer Physics Inc., Herzliya, Israel) used in this study did not have a factory pre-established prediction model to measure the DM of the forage or silage samples at the time of study. Thus, 164 samples (35.4 ± 4.88% DM; ±SD) ranging from 20.7 to 45.8% DM of whole-plant corn silage (WPCS) were collected at the University of Florida Dairy Unit (Gainesville, FL, USA). These samples were selected to represent a wide range of DM concentrations in WPCS, thus enhancing the predictive accuracy of the calibration model developed by SciO. Corn silage samples were collected in duplicate, once a week, by sampling silage piles that were defaced at feed-out. The face of the silo was sampled in the shape of a “W”, with samples taken from an area of approximately one square meter at each point of the “W” and split using a quartering technique before collection. Sampling from multiple piles was essential to ensure independence among the collected samples. The reflectance curve used by pNIRS to predict DM was obtained by scanning WPCS samples allocated into aluminum pans with a sample depth of approximately 5 cm, in five different spots, with two different pNIRS. Subsequently, duplicate 50 g samples were dried for 48 h at 60 °C in a forced-air oven (Heratherm, Thermo Fisher Scientific, Waltham, MA, USA). Using the test model, compared to forced-air oven DM, the SciO Developer license created a prediction model on the SciO cloud. In the SciO calibration model, spectra from repeated scans (n = 5) were averaged, converted to absorbance (log R^−1^), and mean-centered. The calibration was conducted by using partial least squares regression, which is a full-spectrum regression methodology typically used for laboratory calibrations, hence improving the predictive accuracy of the calibration curve. The DM values predicted for pNIRS were obtained using the test model function of the *The Lab* mobile app by the manufacturer, and the same model was used for all measurements (R^2^ = 0.47; RMSE = 3.459). The SciO did not have a WPCF calibration, and WPCF samples were evaluated using a WPCS calibration.

### 2.2. Comparison of DM Measurement Methods in Whole-Plant Corn Forage with Forced-Air Oven Drying as the Reference Method (Experiment 1)

For this experiment, 48 samples of WPCF were collected from two locations during the spring harvesting season of 2018. A total of 25 corn hybrids planted in individual plots were hand-collected. These areas were established for a hybrid performance trial at the University of Florida/IFAS Plant Science Research and Education Unit (Citra, FL, USA). Sampling from multiple plots and corn hybrids was essential to ensure independence among the collected samples. Samples were collected over multiple days, as hybrids varied in relative maturity and other traits. In addition, 23 samples of approximately 1 kg each were collected from 23 trucks (1 sample per truck) unloaded into silos at Full Circle Dairy LLC (Lee, FL, USA). Samples from each truck represented many different hybrids and fields (with fields defined based on the combination of individual center pivots and hybrids) at harvest and were placed into polyethylene bags (ZipLoc, San Diego, CA, USA). All samples were frozen at −20 °C immediately, and after thawing, all DM measurements were conducted on the same day, within two hours, at the University of Florida Department of Animal Science (Gainesville, FL, USA). In this experiment, the treatments included one forced-air oven (average of two replicates), the average of two Koster testers (Koster Crop Tester, Inc., Medina, OH, USA) with one replicate by Koster for a single DM value (34.3 ± 5.68% DM; ±SD), and three pNIRS units (30.5 ± 7.22% DM; ±SD). The forced-air oven was set at 60 °C, and duplicate 50 g samples were weighed after 48 h of drying. Koster testers were used to evaluate duplicate 50 g samples weighed after 30 min and every 10 min thereafter until the sample weight did not change by more than 0.1 g. The pNIRS had internal calibration performed at the beginning of each day with the lens covered. Portable near-infrared reflectance spectrometers were recalibrated any time the units were turned off. To measure DM with pNIRS, thawed samples were placed into small plastic buckets with a sample depth of approximately 10 cm. Samples of approximately 1 kg were scanned, hand-mixed, and scanned again with each pNIRS. This procedure was repeated five times (as required by the manufacturer of *The Lab* for DM prediction). Then, 50 g duplicate subsamples taken using the quartering technique were collected and weighed into aluminum pans (0.13 × 0.10 × 0.05 m) immediately following scanning with pNIRS for drying in both the forced-air oven and Koster testers. After drying, 5 different samples were randomly collected, weighed, and processed immediately to evaluate residual moisture concentration by drying samples from forced-air oven and Koster testers at 105 °C in a forced-air oven for 24 h.

### 2.3. Comparison of Koster Tester and pNIRS Measurement Methods in Whole-Plant Corn Forage (Experiment 2)

In this experiment, 65 WPCF samples were collected during the spring harvest in 2019 from Full Circle Dairy LLC (Lee, FL, USA) and placed into polyethylene bags (ZipLoc, San Diego, CA, USA). Samples were collected as described previously in Experiment 1, except for samples being collected over a 2–3-week period to ensure samples were not collected from the same fields and hybrids (this set of samples represented fields covering approximately 400 hectares). All samples were analyzed at the farm immediately after collection. Due to time restrictions related to harvesting at the farm, the forced-air oven method was not included. Therefore, this trial comprised four treatments: the average of two Koster testers with one replicate by Koster for a single DM value (37.3 ± 4.63% DM; ±SD) and three pNIRS units (33.3 ± 5.74% DM; ±SD). The previously described DM calibration curve provided by SciO from WPCS for the pNIRS was used. The predicted DM values and calibration for pNIRS were obtained as described previously. Subsamples of about 150 g were taken using the quartering technique and placed in aluminum pans (0.22 × 0.11 × 0.06 m) with a sample depth of approximately 7 cm, and each pan was scanned with each of the three pNIRS. This procedure was repeated five times. A Koster tester was used to evaluate 100 g single replicate samples weighed after 40 min and every 5 min thereafter until the sample weight did not change by more than 0.5 g (value used due to lower scale precision). After drying in the Koster tester, 6 different samples were randomly collected, weighed, and processed immediately to evaluate residual moisture concentration, as described previously.

### 2.4. Comparison of DM Measurement Methods in Whole-Plant Corn Silage with Forced-Air Oven Drying as the Reference Method (Experiment 3)

This experiment was conducted with 27 samples of WPCS collected throughout 2019 from three different locations. Fifteen samples were collected from different ag bag silos (3.7 × 152 m) by hand-grabbing samples from each of the ag bag silos at Full Circle Dairy LLC (Lee, FL, USA). Three samples, one sample from each of the three independent ag bags, were collected weekly for 5 weeks. This protocol ensured that a single replicate was collected from each ag bag silo (bags fully fed in 10 d). Three samples were collected from three independent bunker silos at the University of Florida Dairy Unit (Gainesville, FL, USA). The face of the silo was sampled in the shape of a “W”, with samples taken from an area of approximately one square meter at each point of the “W” and split using a quartering technique before collection. Nine samples were collected from laboratory silos from nine independent silage studies, with a single replicate sample taken using the quartering technique from each of these studies conducted at the University of Florida Department of Animal Science (Gainesville, FL, USA) and placed into polyethylene bags (ZipLoc, San Diego, CA, USA). Samples from laboratory silos originated from various hybrids and locations, with each study designed to assess different aspects of silage production (i.e., maturity, microbial inoculation, chop height, chop length, and storage length). All samples were frozen at −20 °C immediately and then thawed later, with DM measurements conducted on the same day, within two hours of thawing. This trial consisted of five treatments: one forced-air oven (the average of two replicates), the average of two Koster testers with one replicate by Koster for a single DM value (33.3 ± 2.68% DM; ±SD), and three pNIRS units (32.9 ± 1.64% DM; ±SD). The previously described DM calibration curve provided by SciO from WPCS for the pNIRS was used. All DM measurements and residual moisture concentration evaluations were conducted as described in the comparison of DM measurement methods in WPCF using forced-air oven as the reference method.

### 2.5. Statistical Analyses

Experiments were analyzed separately using PROC GLIMMIX in SAS 9.3 according to the following model:

$$Y_{ijk}=\mu +T_{i}+\;L_{k}+\;e_{ijk},$$

with $L_{k}\;\approx \;N\;(0,\;\sigma_{Lk}^{2}$) and $e_{ijk}\;\approx \;N\;(0,\;\sigma_{e}^{2}$), where $Y_{ijk}$ is the value of the dependent variable; μ is the overall mean; $T_{i}$ is the fixed effect of treatment (*i* = 1 to 5) for Experiments 1 and 3, whereas $T_{i}$ is the fixed effect of treatment (*i* = 1 to 4) for Experiment 2; $L_{k}$ is the random effect of location (*k* = 1 to 2) for Experiment 1, whereas $L_{k}$ is the random effect of location (*k* = 1 to 3) for Experiment 3; and $e_{ijk}$ is the residual error. *N* stands for Gaussian distribution; $\sigma_{e}^{2}$ is the residual variance; and $\sigma_{Lk}^{2}$ is the variance associated with the random effect of location. Correlations among treatments were assessed using PROC CORR in SAS 9.3, using the Pearson Correlation Coefficients function. For residual moisture concentration data, treatments were considered *i* = 1 to 2 (forced-air oven and Koster tester), but only descriptive statistics were analyzed in Experiment 2, as forced-air oven drying was not tested. Means were determined using the LSMEANS statement. If an overall treatment effect was detected (*p* ≤ 0.05), treatment means were compared using the Bonferroni T-test option. Statistical significance was declared at *p* ≤ 0.05.

For all experiments, assessment of pNIRS quality of prediction was performed. Repeatability and reproducibility were assessed based on evaluations conducted by [23]. To estimate the quality of the pNIRS prediction, the average results from each pNIRS were compared with observed values from forced-air oven and/or Koster tester(s) in each experiment. The goodness-of-fit values of pNIRS predictions were assessed using the coefficient of determination (R^2^) between predicted and observed values. Although R^2^ indicates the degree of association between the predicted values, it does not evaluate the agreement between methods [24]. Therefore, Bland–Altman plots were used for regression analysis to evaluate the relationship between methods. The Bland–Altman plot method determines the bias between methodologies by calculating the mean difference and the 95% CI (mean ± 1.96 SD) of the difference between two methods of measurement [24]. Accuracy was also evaluated based on the concordance correlation coefficient (CCC), mean bias, root of mean square error prediction (RMSEP), and standard error of prediction (SEP).

The CCC is the multiplication of correlation coefficient (r) between observed and predicted values by the bias correction, which indicates how far the regression line deviates from the slope of unity [25]. The RMSEP equation is the square root of mean square error prediction (MSEP), where MSEP is the sum of the squared difference between observed values and model-predicted values divided by the number of observations (n; [26]). The MSEP was decomposed into mean bias, slope bias, and random errors [27].

## 3. Results and Discussion

This current study aimed to assess the DM variation in whole-plant corn because it is the predominant forage used in dairy rations worldwide [28]. Previous work [29] reported that feed components affect nutrient variation of the delivered diets in proportion to the square of the inclusion level and the variability in ingredient composition. Although forages are typically considered ingredients with medium nutrient variability [30], the high inclusion rate considerably impacts dietary nutrient variability. Further research is needed to evaluate pNIRS in other forages and feeds that might be categorized with medium-to-high nutrient variability (i.e., less common byproducts).

### 3.1. Comparison of DM Measurement Methods in Whole-Plant Corn Forage with Forced-Air Oven Drying as the Reference Method (Experiment 1)

**Figure 1:**
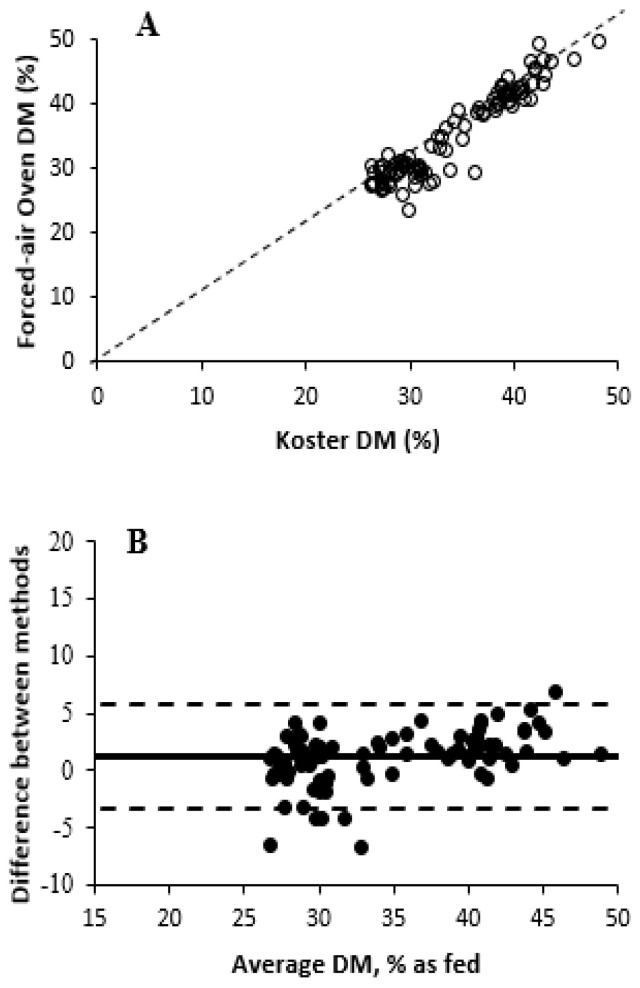
Comparison of dry matter measurement methods in whole-plant corn forage with forced-air oven drying as the reference method (Experiment 1). (**A**) Relationship between forced-air oven dry matter (%) values and Koster tester. Dashed line represents Y = X, if Koster tester was equivalent to forced-air oven values. (**B**) Bland–Altman plot illustrating agreement between forced-air oven and Koster tester prediction. The X-axis presents the average DM concentration between forced-air oven and Koster tester, and the Y-axis presents the difference in DM concentration between forced-air oven and Koster tester (forced-air oven/Koster tester). Central, upper, and lower horizontal lines correspond to the mean (1.14) and higher (5.70) and lower (−3.41) 95% limits of agreement (mean ± 1.96 SD), respectively (SD = 2.31).

**Figure 2:**
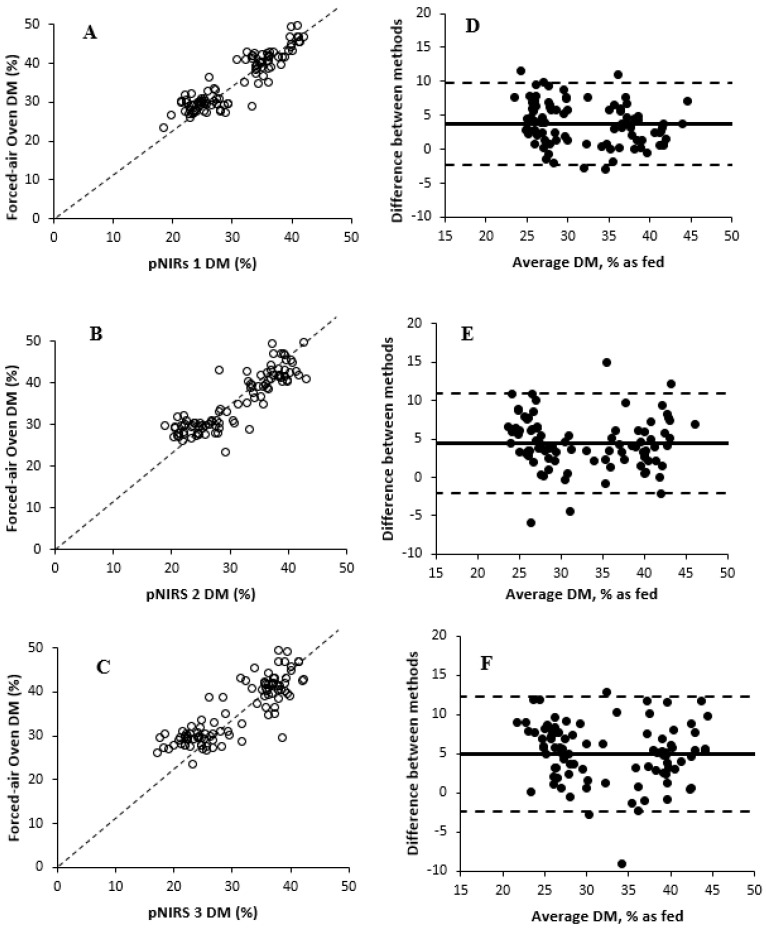
Comparison of dry matter measurement methods in whole-plant corn forage with forced-air oven drying as the reference method (Experiment 1). (**A**–**C**) Relationships between forced-air oven dry matter (%) values and all other pNIRS. Dashed line represents Y = X, if pNIRS were equivalent to forced-air oven values. Bland–Altman plot (Panel (**D**)). The X-axis presents the average DM concentration between forced-air oven and pNIRS 1, and the Y-axis presents the difference in DM concentration between forced-air oven and pNIRS 1 (forced-air oven/pNIRS 1). Central, upper, and lower horizontal lines correspond to the mean (3.75) and higher (9.74) and lower (−2.23) 95% limits of agreement (mean ±1.96 SD), respectively (SD = 3.05). Bland–Altman plot (Panel (**E**)). The X-axis presents the average DM concentration between forced-air oven and pNIRS 2, and the Y-axis presents the difference in DM concentration between forced-air oven and pNIRS 2 (forced-air oven/pNIRS 2). Central, upper, and lower horizontal lines correspond to the mean (4.45) and higher (10.87) and lower (−1.97) 95% limits of agreement (mean ±1.96 SD), respectively (SD = 3.27). Bland–Altman plot (Panel (**F**)). The X-axis presents the average DM concentration between forced-air oven and pNIRS 3, and the Y-axis presents the difference in DM concentration between forced-air oven and pNIRS 3 (forced-air oven/pNIRS 3). Central, upper, and lower horizontal lines correspond to the mean (4.98) and higher (12.35) and lower (−2.39) 95% limits of agreement (mean ± 1.96 SD), respectively (SD = 3.76).

The effects of DM measurement methods are described in Table 1. Based on values obtained from forced-air oven drying, the DM of fresh WPCF samples ranged from 23.4 to 49.6% DM. An overall treatment effect of DM measurement methods was observed (*p ≤* 0.001) for Experiment 1. The average DM of forced-air oven differed (*p =* 0.01) from Koster tester treatment (35.4 vs. 34.3% DM, on average, respectively) and from all three pNIRS units (*p ≤* 0.001; 30.7% DM, on average), with no difference (*p ≥* 0.99) among the pNIRS units (coefficient of variation for pNIRS 1 = 6.13; pNIRS 2 = 7.06; and pNIRS 3 = 6.87). Previous studies have found inaccurate DM values of forage using Koster testers [9,11], but [11] reported that the total error with Koster testers is acceptable for on-farm use. The Koster tester treatment was numerically similar compared with the forced-air oven treatment. Additionally, ref. [31] described that pNIRS DM results differed by approximately 4 percentage units from the forced-air oven treatment, as observed in the results in this experiment. Ref. [32] previously described that a 7-percentage-unit threshold for a weekly range in DM was sufficient to involve rebalancing of dairy diets. In this current study, pNIRS had poor accuracy by underestimating the DM concentration of WPCF, causing a systematic bias that could limit both diet formulation accuracy and the applicability of pNIRS for routine decision making on farms. Differences between pNIRS and other methods may be related to the calibration model being created solely with ensiled samples, whereas the experiment used unfermented samples. The relationships and correlations among treatments are shown in Figure 1 and Figure 2 and Table 2, respectively. All treatments were positively correlated (r ≥ 0.86) with the forced-air oven treatment DM values. Among the pNIRS units, pNIRS 1 showed the highest correlation compared with forced-air oven (r = 0.91, *p* < 0.001), followed by pNIRS 2 (r = 0.89) and pNIRS 3 (r = 0.86). These findings suggest lower robustness of the method, as pNIRS 1 may provide more reliable DM concentration estimates compared with other units evaluated in the study. Small deviations in predicting DM concentration can affect diet formulation or real-time harvesting decisions, thereby influencing the profitability of dairy farms. All pNIRS units were also positively correlated (r ≥ 0.85) with Koster tester treatment DM values. However, the Bland–Altman plots showed greater differences in DM concentration between forced-air oven and pNIRS treatments than Koster testers. Koster testers showed a small negative mean bias, indicating that the Koster tester slightly underestimated DM concentrations compared with forced-air oven. However, some data points were dispersed at higher DM values, suggesting increased variability for WPCS with greater DM concentration. In addition, pNIRS 3 showed greater mean bias and widest 95% limits of agreement, suggesting greater random error and lower precision than both pNIRS 1 and pNIRS 2.

The high correlation between these methods indicates that sample collection was effective and that the scanning technique for pNIRS was consistent [32]. The effects of DM prediction method on residual moisture concentration are presented in Table 3. There was no difference (*p =* 0.68) in residual moisture concentration for forced-air oven and Koster tester treatments (3.2% moisture, on average), which is in agreement with previous reports [11,31], where approximately 3.0%–4.5% residual moisture remained after the completion of these drying methods. The relationships between forced-air oven and Koster tester treatments and those determined by three pNIRS units are shown in Table 4. Koster tester predictions had a higher R^2^, lower mean bias, and higher CCC compared with pNIRS treatment. In addition, the pNIRS DM values for all three devices were lower than the forced-air oven DM value, with numerically similar mean biases among pNIRS units, suggesting that it is possible that the pNIRS units could be as accurate as forced-air oven drying at 60 °C for 48 h if the calibrations were corrected for bias.

### 3.2. Comparison of Koster Tester and pNIRS Measurement Methods in Whole-Plant Corn Forage (Experiment 2)

**Figure 3:**
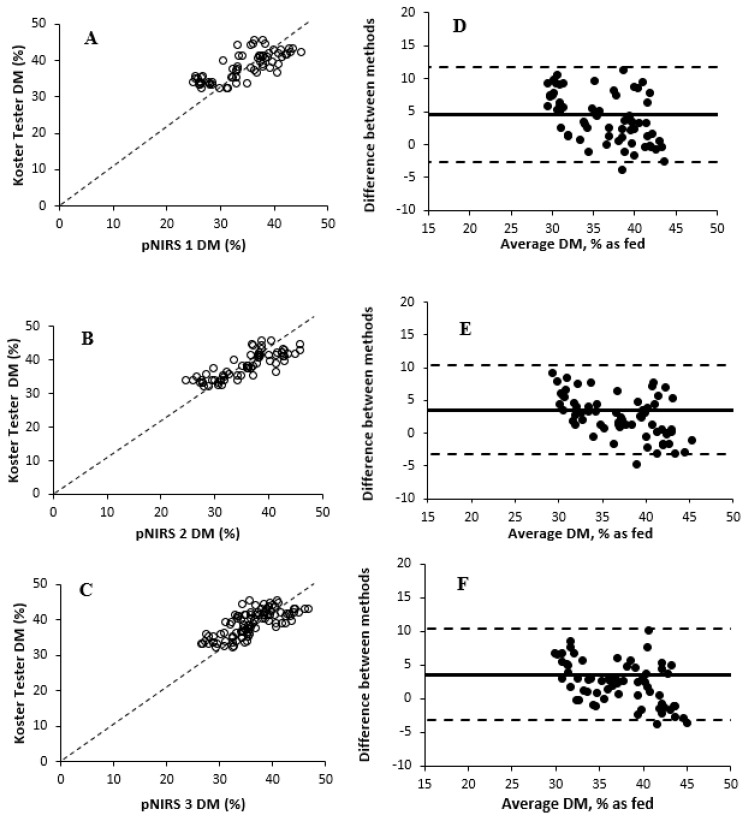
Comparison of dry matter measurement methods in whole-plant corn forage with Koster tester as the reference method (Experiment 2). (**A**–**C**) Relationships between Koster tester dry matter (%) values and all other treatments in Experiment 2. Dashed line represents Y = X, if other treatments were equivalent to Koster tester values. Bland–Altman plot (Panel (**D**)). The X-axis presents the average DM concentration between Koster tester and pNIRS 1, and the Y-axis presents the difference in DM concentration between Koster tester and pNIRS 1 (Koster tester/pNIRS 1). Central, upper, and lower horizontal lines correspond to the mean (4.54) and higher (11.80) and lower (−2.72) 95% limits of agreement (mean ±1.96 SD), respectively (SD = 3.69). Bland–Altman plot (Panel (**E**)). The X-axis presents the average DM concentration between Koster tester and pNIRS 2, and the Y-axis presents the difference in DM concentration between Koster tester and pNIRS 2 (Koster tester/pNIRS 2). Central, upper, and lower horizontal lines correspond to the mean (3.60) and higher (10.44) and lower (−3.23) 95% limits of agreement (mean ±1.96 SD), respectively (SD = 3.49). Bland–Altman plot (Panel (**F**)). The X-axis presents the average DM concentration between Koster tester and pNIRS 3, and the Y-axis presents the difference in DM concentration between Koster tester and pNIRS 3 (Koster tester/pNIRS 3). Central, upper, and lower horizontal lines correspond to the mean (3.56) and higher (10.36) and lower (−3.23) 95% limits of agreement (mean ± 1.96 SD), respectively (SD = 3.47).

The effects of DM measurement methods are reported in Table 5. An overall treatment effect of DM measurement methods was observed (*p* ≤ 0.001) for Experiment 2. Values obtained from Koster testers ranged from 27.2 to 45.7% DM. Koster tester DM determinations differed (*p* ≤ 0.001) from all three pNIRS units (37.2 vs. 33.3% DM, on average, respectively). Conversely, [32] reported that pNIRS overpredicted DM values of corn silage samples and showed lower accuracy compared with other devices (i.e., Aurora). The relationships and correlations among treatments are shown in Figure 3 and Table 6, respectively. All pNIRS units were positively correlated (r ≥ 0.80) with Koster tester treatment, showing a high repeatability and consistency for pNIRS (coefficient of variation for pNIRS 1 = 4.50; pNIRS 2 = 6.33; and pNIRS 3 = 5.68). However, the Bland–Altman plots showed that measurements differed greatly in DM concentration between Koster tester and pNIRS 1 treatments in comparison to others. All pNIRS had negative mean biases, suggesting a tendency to underestimate DM concentration of WPCF compared with Koster testers. Furthermore, systematic deviation and random error were more pronounced in pNIRS 3 due to more dispersed data and broader limits of agreement compared with other units. On average, residual moisture concentration of the Koster tester was 1.7% moisture, as observed in Table 7. The relationships between Koster tester DM determinations and those determined by three pNIRS units are shown in Table 8. pNIRS 1 had a slightly lower R^2^ compared with pNIRS 2 and pNIRS 3 treatments (0.64 vs. 0.70, on average, respectively), with the greatest mean bias and RMSEP (−4.54 and 5.84% DM, respectively) associated with a lower CCC (0.59 vs. 0.68, on average, respectively). The predominance of random error as the main contributor to the MSEP suggests limited repeatability of the model, which could be overcome by using an updated calibration set with samples representing a wider range of composition and physical characteristics. Likewise, ref. [31] reported prediction errors that were associated with pNIRS calibrations, small ranges in forage DM (i.e., 2-percentage-unit difference between the lowest and highest DM values), and the reference method used (forced-air oven drying at 60 °C for 48 h). In addition, although high repeatability might be expected for any method, previous studies [11] reported that electronic testers (i.e., pNIRS units) showed greater variation because these testers use an indirect measurement (reflectance curve) to predict feed DM concentration. There are several factors that could affect the predicted DM concentration, such as the particle size of forage samples, signal to-noise ratio issues, and operator [32].

### 3.3. Comparison of DM Measurement Methods in Whole-Plant Corn Silage with Forced-Air Oven Drying as the Reference Method (Experiment 3)

**Figure 4:**
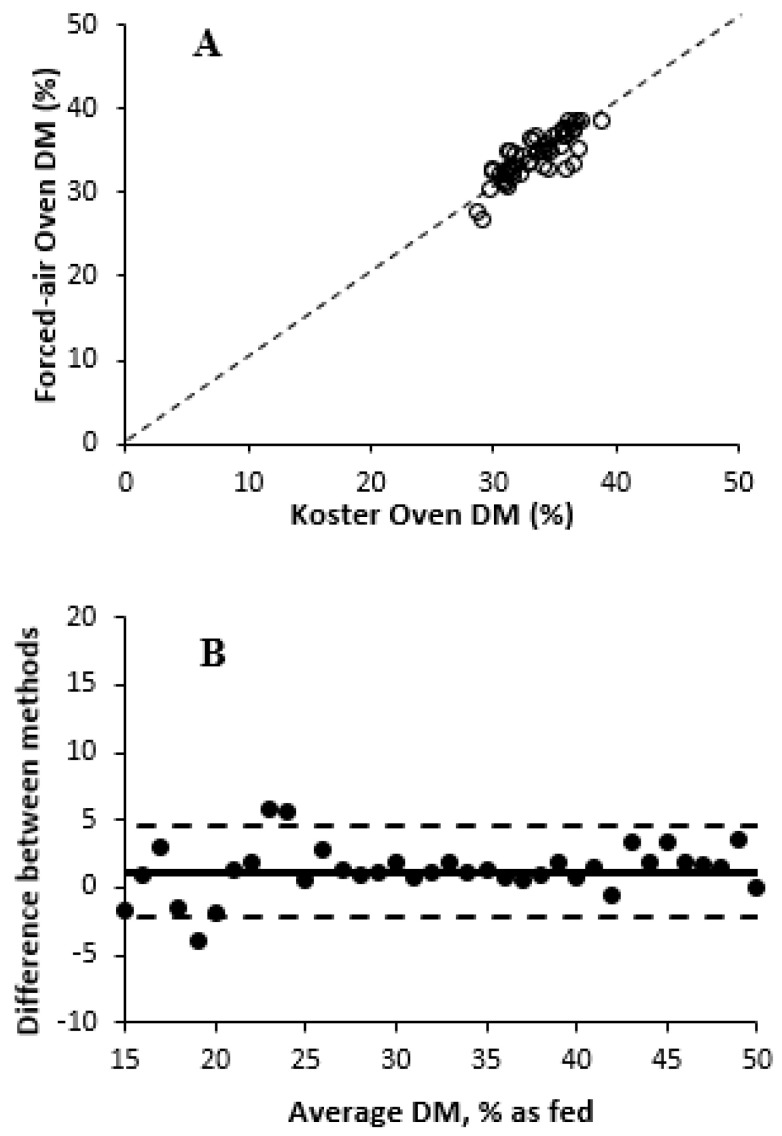
Comparison of dry matter measurement methods in whole-plant corn silage with forced-air oven drying as the reference method (Experiment 3). (**A**) Relationship between forced-air oven dry matter (%) values and Koster tester in Experiment 3. Dashed line represents Y = X, if Koster tester was equivalent to forced-air oven values. (**B**) Bland–Altman plot illustrating agreement between forced-air oven and Koster tester prediction. The X-axis presents the average DM concentration between forced-air oven and Koster tester, and the Y-axis presents the difference in DM concentration between forced-air oven and Koster tester (forced-air oven/Koster tester). Central, upper, and lower horizontal lines correspond to the mean (1.19) and higher (4.53) and lower (−2.15) 95% limits of agreement (mean ± 1.96 SD), respectively (SD = 1.70).

**Figure 5:**
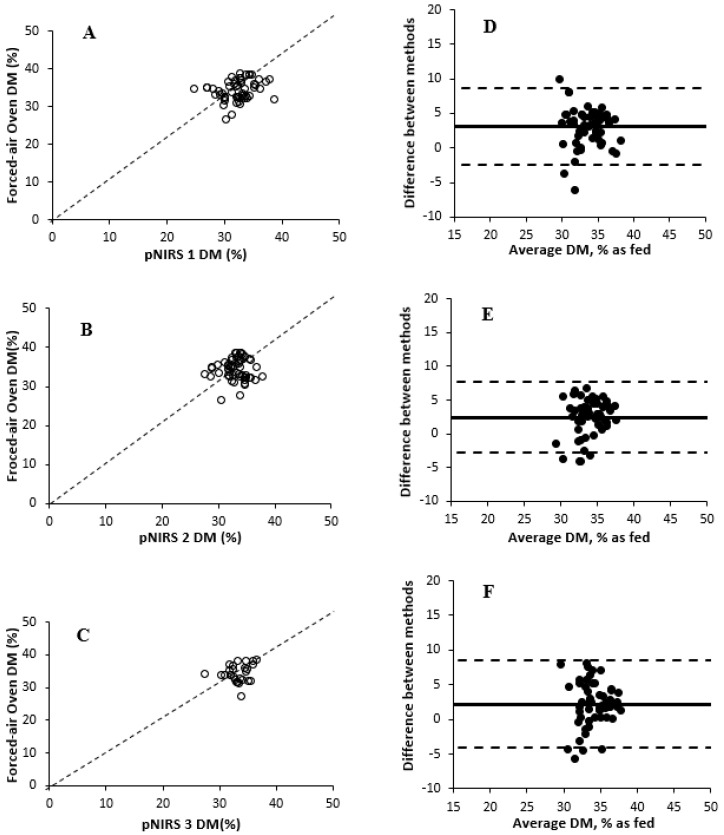
Comparison of dry matter measurement methods in whole-plant corn silage with forced-air oven drying as the reference method (Experiment 3). (**A**–**C**) Relationships between forced-air oven dry matter (%) values and all other pNIRS in Experiment 3. Dashed line represents Y = X, if pNIRS were equivalent to forced-air oven values. Bland–Altman plot (Panel (**D**)). The X-axis presents the average DM concentration between forced-air oven and pNIRS 1, and the Y-axis presents the difference in DM concentration between forced-air oven and pNIRS 1 (forced-air oven/pNIRS 1). Central, upper, and lower horizontal lines correspond to the mean (3.05) and higher (8.61) and lower (−2.51) 95% limits of agreement (mean ±1.96 SD), respectively (SD = 2.84). Bland–Altman plot (Panel (**E**)). The X-axis presents the average DM concentration between forced-air oven and pNIRS 2, and the Y-axis presents the difference in DM concentration between forced-air oven and pNIRS 2 (forced-air oven/pNIRS 2). Central, upper, and lower horizontal lines correspond to the mean (2.44) and higher (7.72) and lower (−2.83) 95% limits of agreement (mean ± 1.96 SD), respectively (SD = 2.69). Bland–Altman plot (Panel (**F**)). The X-axis presents the average DM concentration between forced-air oven and pNIRS 3, and the Y-axis presents the difference in DM concentration between forced-air oven and pNIRS 3 (forced-air oven/pNIRS 3). Central, upper, and lower horizontal lines correspond to the mean (2.20) and higher (8.49) and lower (−4.08) 95% limits of agreement (mean ± 1.96 SD), respectively (SD = 3.20).

An overall treatment effect of DM measurement methods was observed (*p* < 0.001) for Experiment 3. The minimum and maximum DM values for forced-air oven in Experiment 3 were 30.4 and 38.6% DM, respectively, as shown in Table 9. In addition, the forced-air oven DM values were greater than other treatments (35.3 vs. 32.8% DM, on average, respectively). Contrary to previous studies [32], the DM concentration of silages was less variable than the DM concentration of forages in the current experiment, and pNIRS DM values were lower than those of the forced-air oven treatment (coefficient of variation for pNIRS 1 = 3.46; pNIRS 2 = 3.05; and pNIRS 3 = 3.62). It is possible that the number of samples used in the predictive curve for this experiment was not sufficient to account for the heterogeneity of these samples [33]. The relationships and correlations among treatments are described in Figure 4 and Figure 5 and Table 10, respectively. Forced-air oven and Koster tester treatments were correlated (r = 0.85), whereas pNIRS units showed minimal correlations (r ≤ 0.34) with forced-air oven treatment. This lack of correlation was corroborated by the wide variability reported in the Bland–Altman plots. The range in forage DM was narrow, and correlations were most likely affected by pNIRS calibrations. Although Koster testers had small mean bias compared with forced-air oven, greater variability was observed for WPCS samples with lower DM concentration. In addition, all pNIRS underestimated DM concentrations, similar to both Experiments 1 and 2. pNIRS 3 showed the widest 95% limits of agreement and greatest bias compared with other units, indicating its lack of consistency in the study. The effects of DM prediction method on residual moisture concentration are presented in Table 11, with no differences (*p =* 0.92) in residual moisture concentration between forced-air oven and Koster tester treatments (3.1% moisture, on average). This is similar to the value of residual moisture reported in [31], which was 2.8% moisture, on average. The relationships between forced-air oven and Koster tester treatments and those determined by three pNIRS units are shown in Table 12. Koster tester predictions can be considered moderately useful, whereas pNIRS DM predictions showed low success for WPCS DM determination [32]. Although R^2^ is highly dependent on dataset range, pNIRS DM predictions had lower CCC and greater RMSEP compared with forced-air drying treatment.

The lower mean bias of pNIRS units in Experiment 3 compared with Experiments 1 and 2 suggests that better prediction of DM concentration would be observed using a calibration model created with unfermented samples. Likewise, mean bias was also reported by ref. [32] when they used haylage calibrations for TMR samples, which was also associated with reduced precision. The lower mean bias for WPCS compared with WPCF was due to the calibration curve built based on fermented samples, accounting for morphological changes in the silage, which could increase DM variations in the husk, stalk, cob, and kernel material during ensiling [31]. Also, differences between sample types in the predictions of DM concentration could be affected by a variety of factors, including the range of values within sample type and interference due to other constituents (i.e., volatile fatty acids). The corn silage samples that were clearly either underpredicted or overpredicted by pNIRS units in terms of DM concentration tended to originate from variation within locations or among locations.

The accuracy of pNIRS predictions is dependent upon a precise calibration to properly assess variation in DM concentration. Under the conditions of the current study, the results suggest that pNIRS units have room for improvement in accurately and precisely estimating the DM concentration of corn forage samples, while the Koster tester(s) had better performance compared to forced-air oven treatment. For WPCS samples, the DM values obtained from pNIRS units would have greater performance with a greater number of samples to account for the larger variation in the constituents of WPCS.

Although pNIRS is considered a promising technology for real-time forage analysis, there was a significant variability observed in pNIRS predictions, particularly in unfermented samples. In the current study, DM prediction of WPCF was performed using a calibration model developed by SciO from scanned samples of WPCS. [21] also observed that mixed silage calibration was more effective for predicting the DM of haylages than either legume or grass silage calibrations. Likewise, ref. [34] failed to predict the nutritive value of perennial ryegrass due to their calibration equations being built for a forage-type set, different to the one examined. In addition, the prediction of DM values of WPCS in Experiment 3 exhibited lower precision than the Koster tester, likely due to a much smaller NIR scanning range associated with limited number of samples. A consistent underprediction of DM values was observed across all three experiments (~4 percentage units), and further adjustments in the prediction curve could significantly enhance the performance of these devices [31,32]. Normally, NIR estimation of DM should be ± 3.5 percentage units or smaller, thereby becoming potentially valuable for daily rebalancing of dairy diets [21]. The predominance of random error as the main contributor to the MSEP suggests limited repeatability of the model. Strategies to improve repeatability include standardizing sample preparation (i.e., homogenization and presentation), performing multiple scans per sample and averaging results, controlling environmental conditions during scanning (i.e., temperature, humidity, ambient light), and applying spectral pre-treatments such as standard normal variate (SNV) or Savitzky–Golay derivatives to reduce instrumental noise. Additionally, expanding and updating the calibration set with samples representing a wider range of composition and physical characteristics may enhance model robustness. Routine maintenance and calibration of the NIR instrument are also essential to ensure spectral stability over time. Furthermore, for WPCF samples, the WPCS calibration developed by SciO is unlikely to provide the most accurate results. Further research and calibration are necessary to enhance the precision and accuracy of pNIRS units, especially for diverse forage materials. Additionally, further investigation is warranted to determine the efficacy of pNIRS units for the nutrient value of corn forage and silage samples.

## 4. Conclusions

Across the three experiments, forced-air oven and Koster tester treatments provided consistent DM values, while pNIRS units tended to underpredict DM values. In addition, the predominance of random error suggests low repeatability of pNIRS units. Differences between pNIRS and other methods may also be related to the calibration model being created solely with ensiled samples, whereas the experiment used unfermented samples. Thus, expanding and updating the calibration set with samples representing a wider range of composition and physical characteristics may enhance model robustness. Therefore, analysis of whole-plant corn forage requires further calibration adjustments. Further research and calibration are necessary to enhance the precision and accuracy of pNIRS units, especially for diverse forage materials.

## Figures and Tables

**Table 1:** Effects of dry matter measurement methods in whole-plant corn forage with forced-air oven drying as the reference method (Experiment 1).

Measurement Method	Mean	Standard Deviation	Minimum	Maximum
Forced-air oven ^1^	35.4 ^a^	6.74	23.4	49.6
Koster testers ^2^	34.3 ^b^	5.68	26.3	48.1
pNIRS 1 ^3^	30.6 ^c^	6.41	18.4	42.0
pNIRS 2	31.0 ^c^	6.92	18.8	43.0
pNIRS 3	30.5 ^c^	7.22	17.2	42.2

^abc^ Means within columns with different superscripts differ (*p* ≤ 0.05). ^1^ Samples dried at 60 °C for 48 h. ^2^ Samples dried for 30 min, then weighed every 10 min thereafter until the sample weight did not change by more than 0.1 g. ^3^ pNIRS: Portable near-infrared reflectance spectrometer.

**Table 2:** Correlation ^1^ of dry matter measurement methods in whole-plant corn forage with forced-air oven drying as the reference method (Experiment 1).

	Forced-Air Oven	Koster Testers	pNIRS 1	pNIRS 2	pNIRS 3
Forced-air oven	1.00	0.94	0.91	0.89	0.86
		<0.001	<0.001	<0.001	<0.001
Koster testers		1.00	0.88	0.88	0.85
			<0.001	<0.001	<0.001
pNIRS 1 ^2^			1.00	0.85	0.85
				<0.001	<0.001
pNIRS 2				1.00	0.86
					<0.001
pNIRS 3					1.00

^1^ Results presented as r over corresponding *p*-value; ^2^ pNIRS: Portable near-infrared reflectance spectrometer.

**Table 3:** Effects of dry matter measurement methods in whole-plant corn forage with forced-air oven drying as the reference method (Experiment 1) on residual moisture concentration (%).

Drying Method	Residual Moisture ^1^	Standard Error	Minimum	Maximum
Forced-air oven	3.4%	0.79	2.70	4.16
Koster testers	2.9%	0.79	0.99	6.15

^1^ Samples dried at 105 °C for 24 h.

**Table 4:** Evaluation of predicted dry matter concentration of whole-plant corn forage by Koster tester and pNIRS compared to observed values from forced-air oven drying as the reference method (Experiment 1; n = 48).

Item	R^2^	Mean Bias (% DM)	CCC ^1^	RMSEP (% DM) ^2^	SEP (% DM) ^3^	MSEP Decomposition (%) ^4^		
						Mean Bias	Slope	Random Errors
Koster testers ^5^	0.89	−1.14	0.92	2.57	2.26	19.6	7.20	73.2
pNIRS 1 ^6^	0.84	−4.90	0.72	5.59	2.70	76.3	0.20	23.5
pNIRS 2	0.79	−4.44	0.73	5.52	3.28	65.1	3.00	31.9
pNIRS 3	0.74	−4.97	0.68	6.23	3.61	63.9	5.40	30.7

^1^ CCC = concordance correlation coefficient; ^2^ RMSEP = root mean square error of prediction; ^3^ SEP = standard error of prediction; ^4^ MSEP = mean square error of prediction; ^5^ Samples dried for 30 min, then weighed every 10 min thereafter until the sample weight did not change by more than 0.1 g; ^6^ pNIRS = portable near-infrared reflectance spectrometer.

**Table 5:** Effects of dry matter measurement methods in whole-plant corn forage with Koster tester as the reference method (Experiment 2).

Measurement Method	Mean	Standard Deviation	Minimum	Maximum
Koster testers ^1^	37.2 ^a^	4.84	27.2	45.7
pNIRS 1 ^2^	32.7 ^c^	6.19	17.2	44.9
pNIRS 2	33.7 ^b^	6.23	14.6	45.9
pNIRS 3	33.6 ^b^	6.30	15.7	46.8

^abc^ Means within columns with different superscripts differ (*p* ≤ 0.05); ^1^ Samples dried for 40 min, then weighed every 10 min thereafter until the sample weight did not change by more than 0.5 g; ^2^ pNIRS: Portable near-infrared reflectance spectrometer.

**Table 6:** Correlation ^1^ of dry matter measurement methods in whole-plant corn forage with Koster tester as the reference method (Experiment 2).

	Koster Testers	pNIRS 1	pNIRS 2	pNIRS 3
Koster testers	1.00	0.80	0.83	0.84
		<0.001	<0.001	<0.001
pNIRS 1 ^2^		1.00	0.89	0.89
			<0.001	<0.001
pNIRS 2			1.00	0.89
				<0.001
pNIRS 3				1.00

^1^ Results presented as r over corresponding *p*-value; ^2^ pNIRS: Portable near-infrared reflectance spectrometer.

**Table 7:** Effects of dry matter measurement methods in whole-plant corn forage with Koster tester as the reference method (Experiment 2) on residual moisture concentration (%).

Drying Method	Residual Moisture ^1^	Standard Error	Minimum	Maximum
Forced-air oven	1.7%	0.28	0.95	2.89

^1^ Samples dried at 105 °C for 24 h.

**Table 8:** Evaluation of predicted dry matter concentration of whole-plant corn forage by pNIRS compared to observed values from Koster tester as the reference method (Experiment 2; n = 65).

Item	R^2^	Mean Bias (% DM)	CCC ^1^	RMSEP (% DM) ^2^	SEP (% DM) ^3^	MSEP Decomposition (%) ^4^		
						Mean Bias	Slope	Random Errors
pNIRS 1 ^5^	0.64	−4.54	0.59	5.84	3.67	60.2	15.6	24.2
pNIRS 2	0.69	−3.60	0.67	4.99	3.46	51.8	19.5	28.7
pNIRS 3	0.70	−3.56	0.68	4.95	3.44	51.6	20.5	27.9

^1^ CCC = concordance correlation coefficient; ^2^ RMSEP = root mean square error of prediction; ^3^ SEP = standard error of prediction; ^4^ MSEP = mean square error of prediction; ^5^ pNIRS = portable near-infrared reflectance spectrometer.

**Table 9:** Evaluation of predicted dry matter concentration of whole-plant corn silage by Koster tester and pNIRS compared to observed values from forced-air oven drying as the reference method (Experiment 3).

Measurement Method	Mean	Standard Deviation	Minimum	Maximum
Forced-air oven ^1^	35.3 ^a^	2.11	30.4	38.6
Koster testers ^2^	34.0 ^b^	2.06	29.8	37.2
pNIRS 1 ^3^	31.9 ^c^	2.91	24.6	37.9
pNIRS 2	32.5 ^c^	2.02	27.6	35.7
pNIRS 3	32.7 ^b,c^	2.53	25.7	37.1

^abc^ Means within columns with different superscripts differ (*p* ≤ 0.05); ^1^ Samples dried at 60 °C for 48 h; ^2^ Samples dried for 30 min, then weighed every 10 min thereafter until the sample weight did not change by more than 0.1 g;^3^ pNIRS: Portable near-infrared reflectance spectrometer.

**Table 10:** Correlation ^1^ of dry matter measurement methods in whole-plant corn silage with forced-air oven drying as the reference method (Experiment 3).

	Forced-Air Oven	Koster Testers	pNIRS 1	pNIRS 2	pNIRS 3
Forced-air oven	1.00	0.85	0.43	0.14	0.34
		<0.001	0.02	0.45	0.06
Koster testers		1.00	0.30	0.16	0.19
			0.11	0.39	0.31
pNIRS 1 ^2^			1.00	0.59	0.56
				0.001	0.001
pNIRS 2				1.00	0.56
					0.001
pNIRS 3					1.00

^1^ Results presented as r over the corresponding *p*-value; ^2^ pNIRS: Portable near-infrared reflectance spectrometer.

**Table 11:** Effects of dry matter measurement methods in whole-plant corn silage with forced-air oven drying as the reference method (Experiment 3) on residual moisture concentration (%).

Drying Method	Residual Moisture ^1^	Standard Error	Minimum	Maximum
Forced-air oven	3.1%	0.72	2.20	4.28
Koster testers	3.0%	0.72	1.05	6.53

^1^ Samples dried at 105 °C for 24 h.

**Table 12:** Evaluation of predicted dry matter concentration of whole-plant corn silage by Koster tester and pNIRS compared to observed values from forced-air oven drying as the reference method (Experiment 3; n = 27).

Item	R^2^	Mean Bias	CCC ^1^	RMSEP (% DM) ^2^	SEP (% DM) ^3^	MSEP Decomposition (%) ^4^		
						Mean Bias	Slope	Random Errors
Koster testers ^5^	0.72	−1.30	0.71	1.73	1.14	56.0	2.50	41.5
pNIRS 1 ^6^	0.19	−3.41	0.22	4.38	2.75	60.5	21.5	18.0
pNIRS 2	0.02	−2.76	0.08	3.85	2.68	51.0	20.4	28.6
pNIRS 3	0.12	−2.55	0.21	3.71	2.69	47.4	24.7	27.9

^1^ CCC = concordance correlation coefficient; ^2^ RMSEP = root mean square error of prediction; ^3^ SEP = standard error of prediction; ^4^ MSEP = mean square error of prediction; ^5^ Samples dried for 30 min, then weighed every 10 min thereafter until the sample weight did not change by more than 0.1 g; ^6^ pNIRS = portable near-infrared reflectance spectrometer.

## Data Availability

The original contributions presented in this study are included in the article. Further inquiries can be directed to the corresponding author(s).

## Abbreviations

CCC, concordance correlation coefficient; DM, dry matter; MSEP, mean square error prediction; pNIRS, portable near-infrared spectrometer; RMSEP, root of mean square error prediction; SEP, standard error of prediction; WPCF, whole-plant corn forage; WPCS, whole-plant corn silage.
